# Polymorphisms of *PTPN11* gene could influence serum lipid levels in a sex-specific pattern

**DOI:** 10.1186/1476-511X-12-72

**Published:** 2013-05-14

**Authors:** Zhi-Fang Jia, Xue-Yuan Cao, Dong-Hui Cao, Fei Kong, Punyaram Kharbuja, Jing Jiang

**Affiliations:** 1 Division of Clinical Epidemiology, First Hospital of Jilin University, Changchun 130021, China; 2 Department of Gastrointestinal Surgery, First Hospital of Jilin University, Changchun 130021, China

**Keywords:** *PTPN11*, Single nucleotide polymorphism (SNP), Triglyceride (TG), Total cholesterol (TC), Low-density lipoprotein cholesterol (LDL-C), High-density lipoprotein cholesterol (HDL-C)

## Abstract

**Background:**

Previous studies have reported that different genotypes of *PTPN11* gene (protein tyrosine phosphatase, non-receptor 11) were associated with different levels of serum lipids. The aim of this study was to explore the relationship between single nucleotide polymorphisms (SNPs) of *PTPN11* and serum lipids in Northeast Chinese.

**Methods:**

A total of 1003 subjects, 584 males and 419 females, were included in the study and their serum lipids were determined. Five htSNPs (rs2301756, rs12423190, rs12229892, rs7958372 and rs4767860) of *PTPN11* gene were genotyped using TaqMan assay method.

**Results:**

All of the five SNPs were in Hardy-Weinberg equilibrium. The male subjects had higher triglyceride (TG), higher low-density lipoprotein cholesterol (LDL-C) and lower high-density lipoprotein cholesterol (HDL-C) level than females. In males, rs4767860 was found to be associated with serum TG and total cholesterol (TC) levels and rs12229892 was associated with TC level. However, these significant associations could not be observed in females. In females, rs2301756 was found to be associated with TG and rs7958372 was associated with LDL-C level. Haplotype analysis showed that the GCGTG haplotype was associated with slightly higher TG level and ATGCG with higher TC level.

**Conclusions:**

SNPs of *PTPN11* may play a role in serum lipids in a sex-specific pattern. However, more studies are needed to confirm the conclusion and explore the underlying mechanism.

## Introduction

Dyslipidemia such as the increased levels of total cholesterol (TC), triglyceride (TG) or the decreased level of high-density lipoprotein cholesterol (HDL-C) has been concluded to be involved in the higher risk of cardiac-cerebral vascular disease [1]–[3] and has become a serious public health problem [4]. It is a complex trait that many factors, environmental and genetic [5,6], have been reported to be associated with it. However, these factors could only explain part of the total variance, and more factors need to be identified.

Src homology-2 domain-containing protein tyrosine phosphatase 2 (SHP2), a ubiquitously expressed protein tyrosine phosphatase, plays an essential role in many cell signaling events such as metabolic control and transcription regulation [7,8]. SHP2 could regulate the apoB (apolipoprotein B) secretion in insulin-dependent pattern via phosphatidylinositol 3′-kinase [9,10]. SHP2 activity was associated with the expression of the fatty acid-metabolizing enzyme Acyl-CoA synthetase 4 (ACSL4) [11] and the synthesis of steroid [12]. SHP2 deletion mice could develop a profile of higher serum levels of cholesterol, TG, and low-density lipoprotein [8]. Single nucleotide polymorphisms (SNPs) of protein tyrosine phosphatase, non-receptor 11 (*PTPN11*) gene, which encodes SHP2, may be associated with serum lipid levels via changing the activity of SHP2 on lipometabolism.

Jamshidi et al. first reported that one of the tagging SNPs of the *PTPN11* gene, rs11066320, was associated with serum low-density lipoprotein cholesterol (LDL-C) level in normal Caucasian female twins [13] and Lu et al. reported that rs11066322 was associated with plasma HDL-C level. The data from Hapmap database show that variants of *PTPN11* gene present great varieties in different ethnicities. The role of *PTPN11* gene on lipid profile has not been described in Chinese so far. The aim of this study was to explore the association of tagging SNPs of *PTPN11* gene and lipid levels in Chinese normal people.

## Methods

### Subjects

From January to December 2009, people who attended the physical examination center of the First Hospital of Jilin University were invited to the study. A total of 1080 persons signed the informed consent and agreed to participate in this study. Subjects who had been taking lipid-lowing medication were excluded from the analysis (*n* = 73). At last, 1003 subjects, 584 males and 419 females, were included in the analysis. The range of age was from 35 to 79 years, with a median of 49 years. This study protocol was approved by the ethics committee of the First Hospital of Jilin University.

Venous blood samples were obtained from all subjects after overnight fasting. The levels of serum TC, TG, HDL-C and LDL-C were determined by enzymatic methods in an autoanalyzer (Type 7600; Hitachi Ltd., Japan) in our Clinical Laboratory Center. The inter-day coefficient variations (CV) of the two distinct analyte levels (Bio-Rad, USA) of the lab were 3.17% and 3.90% for TC, 2.74% and 2.64% for TG, 3.85% and 4.08% for HDL-C, 3.72% and 3.37% for LDL-C during the researching period.

### Tagging SNPs selection and genotyping

SNP tagging was to identify a set of SNPs that efficiently tags all known SNPs. Haplotype tagging SNPs (htSNPs) were selected from the Han Chinese data in the HapMap Project (06-02-2009 HapMap) using the SNPbrowser™ Software v4.0 to capture SNPs with a minimum minor allele frequency (MAF) of 0.05 with a pair-wise r square of 0.8 or greater [14]. There were nine SNPs at MAF > 0.05 in the *PTPN11* gene in Chinese on HapMap, all of which were located in non-coding regions. Five SNPs, rs2301756, rs12423190, rs12229892, rs7958372 and rs4767860, were selected as htSNPs for further study.

Genomic DNA was extracted from whole blood following the protocols provided by the manufacturer (Axygen, USA). Genotypes of each SNP were determined using TaqMan SNP genotying assays (Applied Biosystems, USA) and the detailed process of polymerase chain reaction (PCR) was described elsewhere [15]. The amplified products of PCR were read on ABI PRISM 7900 Sequence Detector in the end-point mode and genotypes were identified using the Allelic Discrimination Sequence Detector Software V2.3.

### Statistical analysis

Categorical data were described as frequency and percentage and compared using *χ*^2^ test or Fisher exact test when appropriate. Continuous variables were summarized as median (25th to 75th percentiles) and compared by Kraskal-Wallis test among groups. The frequencies of genotypes of each SNP were determined via direct counting and deviation from Hardy-Weinberg equilibrium was assessed by a goodness-of-fit *χ*^2^ test. Levels of TC, TG, HDL-C and LDL-C were transformed to their logarithms to improve the normality of distribution. Associations of the SNPs and lipid levels were assessed using analysis of covariance within each gender type, adjusted for age, body mass index (BMI) and waist circumference. The above analyses were performed in SAS 9.1.3 software (SAS Institute Inc, USA). For haplotypes with frequencies >1%, their associations with lipids were assessed compared to the most common haplotype using the linear regression model with the HAPSTAT software 3.0 [16]. The statistical significance was *P* < 0.05.

## Results

The baseline characteristics of the subjects are shown in Table 1. The body mass index (BMI) was higher than 24.0 Kg/m^2^ in half of the subjects (the median value of BMI was 24.0 Kg/m^2^, with a quartile range from 21.9 to 26.1 Kg/m^2^). No difference was observed between males and females in terms of age, but BMI and waist circumference were higher in males than in females.

**Table 1:** Characteristics of subjects included

	**All (*n*** **= 1003)**	**Male (*n*** **= 584)**	**Female (*n*** **= 419)**	***P***
Age (year)	49 (45–55)	49 (45–54)	48 (44–56)	0.640
Waist (cm)	85 (77–92)	90 (84–94)	77 (72–83)	<0.001
BMI (Kg/m^2^)	24.0 (21.9–26.2)	24.9 (23.0–26.7)	22.6 (20.7–24.9)	<0.001
TG (mmol/L)	1.44 (0.98–2.12)	1.61 (1.16–2.44)	1.21 (0.84–1.73)	<0.001
TC (mmol/L)	5.04 (4.49–5.66)	5.03 (4.48–5.68)	5.09 (4.52–5.64)	0.669
HDL-C (mmol/L)	1.33 (1.15–1.56)	1.27 (1.10–1.45)	1.48 (1.27–1.70)	<0.001
LDL –C (mmol/L)	3.09 (2.63–3.60)	3.10 (2.69–3.63)	3.00 (2.53–3.54)	<0.001
rs2301756				
GG	750 (74.8%)	439 (75.2%)	311 (74.2%)	0.625
GA	232 (23.1%)	131 (22.4%)	101 (24.1%)	
AA	21 (2.1%)	14 (2.4%)	7 (1.7%)	
rs12423190				
TT	515 (51.3%)	304 (52.0%)	211 (50.4%)	0.782
TC	399 (39.8%)	227 (38.9%)	172 (41.0%)	
CC	89 (8.9%)	53 (9.1%)	36 (8.6%)	
rs12229892				
GG	342 (34.1%)	189 (32.4%)	153 (36.5%)	0.271
GA	485 (48.4%)	285 (48.8%)	200 (47.7%)	
AA	176 (17.5%)	110 (18.8%)	66 (15.8%)	
rs7958372				
TT	751 (74.9%)	439 (75.2%)	312 (74.5%)	0.962
TC	235 (23.4%)	135 (23.1%)	100 (23.9%)	
CC	17 (1.7%)	10 (1.7%)	7 (1.7%)	
rs4767860				
AA	335 (33.4%)	198 (33.9%)	137 (32.7%)	0.666
GA	480 (47.9%)	282 (48.3%)	198 (47.3%)	
GG	188 (18.7%)	104 (17.8%)	84 (20.0%)	

Unless indicated, data were described as median (Q1–Q3).

The linkage disequilibrium structure of the five SNPs studied, rs2301756, rs12423190, rs12229892, rs7958372 and rs4767860 is presented in Table 2. They were all in linkage disequilibrium, though to different extents. All of the five SNPs were in Hardy-Weinberg equilibrium (*P* = 0.540, 0.354, 0.778, 0.858, 0.489, respectively). There were no significant differences in the distribution of genotypes between males and females (Table 1). And no differences were observed among genotypes of each SNP in terms of age, sex, BMI and waist circumference (data were not shown).

**Table 2:** **The linkage disequilibrium coefficient (Lewontin’sD’ and r**^**2**^**) between SNPs of PTPN11**

	**rs2301756**	**rs12423190**	**rs12229892**	**rs7958372**	**rs4767860**
rs2301756	--	0.038	0.103	0.871	0.194
rs12423190	0.774	--	0.289	0.055	0.509
rs12229892	0.953	1.000	--	0.107	0.512
rs7958372	0.944	0.937	0.984	--	0.201
rs4767860	0.956	0.968	0.980	0.982	--

Values on the left of “--” were Lewontin’s D’ coefficients and on the right were *r*^2^.

As lipid levels of males were different from those of females, except for cholesterol (Table 1), separate analyses were performed on the association of lipid levels and SNPs.

In males, the median serum level of TG was 1.61 mmol/L, with a quartile range 1.16–2.44 mmol/L. Rs4767860 and rs12229892 were observed to be associated with TG level after controlling for the effects of age, waist circumference and BMI in male subjects. The genotype GG or GA of rs4767860 was found to be with higher TG level compared to the most common genotype AA (*P* = 0.028, 0.024, respectively), and genotype AA of rs12229892 was associated with lower TG level compared to genotype GG (*P* = 0.009, Table 3). The median level of TC was 5.03 mmol/L, and subjects bearing GG genotype of rs4767860, were found to have slightly higher serum TC compared to subjects with genotype AA (5.13 *v*.*s*. 4.98 mmol/L, *P* = 0.021) in males. The median levels of HDL-C and LDL-C were 1.27 mmol/L and 3.10 mmol/L, respectively, and no SNP was found to be related to them.

**Table 3:** **Associations between SNPs of *PTNP11 *and lipid levels stratified by gender**

	**Frequency (%)**	**TG**		**TC**		**HDL-C**		**LDL-C**	
		**Median (Q1–Q3)**	***P***	**Median (Q1–Q3)**	***P***	**Median (Q1–Q3)**	***P***	**Median (Q1–Q3)**	***P***
Male (*n* = 584)									
rs2301756									
GG	439 (75.2)	1.59 (1.09–2.37)	–	5.02 (4.45–5.63)	–	1.25 (1.10–1.43)	–	3.10 (2.68–3.61)	–
GA	131 (22.4)	1.63 (1.20–2.47)	0.319	5.07 (4.52–5.84)	0.161	1.30 (1.10–1.47)	0.164	3.10 (2.68–3.74)	0.236
AA	14 (2.4)	1.74 (1.19–2.85)	0.516	5.03 (4.76–5.32)	0.877	1.30 (1.12–1.60)	0.572	2.98 (2.75–3.65)	0.969
rs12423190									
TT	304 (52.0)	1.57 (1.08–2.44)	–	4.99 (4.46–5.57)	–	1.27 (1.10–1.47)	–	3.10 (2.65–3.62)	–
TC	227 (38.9)	1.67 (1.26–2.33)	0.184	5.10 (4.49–5.74)	0.411	1.26 (1.09–1.42)	0.309	3.14 (2.70–3.63)	0.380
CC	53 (9.1)	1.64 (1.08–2.68)	0.120	5.07 (4.49–5.76)	0.219	1.30 (1.07–1.51)	0.203	3.00 (2.72–3.70)	0.595
rs12229892									
GG	189 (32.4)	1.67 (1.21–2.53)	–	5.13 (4.53–5.72)	–	1.30 (1.10–1.47)	–	3.11 (2.70–3.67)	–
GA	285 (48.8)	1.61 (1.20–2.45)	0.405	5.02 (4.49–5.64)	0.106	1.23 (1.10–1.41)	0.988	3.14 (2.67–3.66)	0.386
AA	110 (18.8)	1.41 (0.96–2.16)	**0.009**	4.96 (4.35–5.50)	0.118	1.29 (1.14–1.49)	0.562	3.08 (2.64–3.57)	0.354
rs7958372									
TT	439 (75.2)	1.59 (1.10–2.37)	–	5.03 (4.46–5.64)	–	1.25 (1.10–1.43)	–	3.10 (2.69–3.61)	–
TC	135 (23.1)	1.64 (1.20–2.47)	0.351	5.05 (4.54–5.77)	0.200	1.30 (1.10–1.47)	0.197	3.10 (2.68–3.74)	0.260
CC	10 (1.7)	1.89 (1.22–2.85)	0.619	4.86 (4.56–5.28)	0.648	1.30 (1.12–1.45)	0.615	2.94 (2.61–3.65)	0.535
rs4767860									
AA	198 (33.9)	1.53 (1.02–2.37)	–	4.98 (4.42–5.51)	–	1.25 (1.10–1.45)	–	3.10 (2.60–3.60)	–
GA	282 (48.3)	1.63 (1.20–2.41)	**0.024**	5.03 (4.48–5.68)	0.185	1.28 (1.09–1.43)	0.973	3.10 (2.67–3.60)	0.252
GG	104 (17.8)	1.64 (1.22–2.64)	**0.028**	5.13 (4.56–5.80)	**0.021**	1.30 (1.10–1.47)	0.598	3.15 (2.75–3.71)	0.084
Female (*n* = 419)									
rs2301756									
GG	311 (74.2)	1.19 (0.83–1.71)	–	4.94 (4.49–5.60)	–	1.48 (1.27–1.69)	–	2.98 (2.50–3.43)	–
GA	101 (24.1)	1.25 (0.85–1.85)	0.780	5.23 (4.72–5.87)	0.071	1.51 (1.27–1.76)	0.283	3.12 (2.66–3.69)	0.083
AA	7 (1.7)	2.33 (1.14–3.20)	**0.005**	5.02 (4.53–6.20)	0.300	1.37 (1.21–1.59)	0.605	3.16 (2.42–3.84)	0.555
rs12423190									
TT	211 (50.4)	1.25 (0.82–1.75)	–	5.03 (4.53–5.67)	–	1.50 (1.27–1.71)	–	3.04 (2.57–3.56)	–
TC	172 (41.0)	1.18 (0.87–1.64)	0.592	5.12 (4.47–5.68)	0.709	1.46 (1.26–1.66)	0.149	3.00 (2.47–3.56)	0.759
CC	36 (8.6)	1.10 (0.80–1.99)	0.388	5.00 (4.64–5.32)	0.877	1.40 (1.22–1.74)	0.184	2.97 (2.52–3.35)	0.895
rs12229892									
GG	153 (36.5)	1.21 (0.85–1.71)	–	5.11 (4.57–5.64)	–	1.48 (1.26–1.70)	–	3.00 (2.65–3.48)	–
GA	200 (47.7)	1.23 (0.86–1.74)	0.497	5.04 (4.45–5.67)	0.599	1.45 (1.27–1.68)	0.880	3.00 (2.51–3.60)	0.873
AA	66 (15.8)	1.19 (0.80–1.68)	0.232	5.07 (4.60–5.61)	0.463	1.53 (1.30–1.77)	0.128	3.07 (2.67–3.40)	0.526
rs7958372									
TT	312 (74.5)	1.19 (0.83–1.71)	–	4.95 (4.50–5.60)	–	1.48 (1.27–1.69)	–	2.98 (2.51–3.44)	–
TC	100 (23.9)	1.25 (0.87–1.88)	0.787	5.22 (4.67–5.93)	0.090	1.52 (1.26–1.76)	0.319	3.10 (2.62–3.63)	0.278
CC	7 (1.7)	1.92 (0.85–2.33)	0.201	5.02 (4.68–6.20)	0.183	1.31 (1.21–1.59)	0.418	3.48 (3.16–4.06)	**0.019**
rs4767861									
AA	137 (32.7)	1.23 (0.81–1.68)	–	4.94 (4.53–5.61)	–	1.51 (1.30–1.70)	–	3.00 (2.57–3.40)	–
GA	198 (47.3)	1.21 (0.86–1.79)	0.474	5.11 (4.45–5.64)	0.515	1.45 (1.24–1.70)	0.113	2.96 (2.46–3.54)	0.600
GG	84 (20.0)	1.21 (0.84–1.91)	0.134	5.16 (4.68–5.87)	0.123	1.41 (1.26–1.67)	0.236	3.13 (2.68–3.58)	0.132

Differences between genotype groups were determined using analysis of covariance within each gender type, adjusted for age, BMI and waist circumference. *P* value in bold indicated the difference was significant comparing to the reference group (*P*<0.05).

In females, however, the results were different. Female subjects had lower TG (1.21 *v*.*s*. 1.61 mmol/l), lower LDL-C (3.00 *v*.*s*. 3.10 mmol/L) and higher HDL-C (1.48 *v*.*s*. 1.27 mmol/L) level than males. The SNPs which were found to be significantly associated with TC or TG level in males could not be repeated in females. However, two other SNPs, rs2301756 and rs7958372, were found to be significantly associated with lipid level in females. The AA genotype of rs2301756 (*P* = 0.005) was found to be associated with higher serum TG level and the CC genotype of rs7958372 (*P* = 0.019) was associated with higher LDL-C level when compared to their most common genotype (Table 3). None of the five SNPs was observed to be associated with TC or HDL-C level.

Because of the linkage disequilibrium, 18 haplotypes were observed using HAPSTAT software which estimated haplotype frequencies based on an EM algorithm and only four of them had the frequencies greater than 1% (Table 4). The GCGTG haplotype, with an estimated frequency of 27.75%, was found to be significantly associated with the increased level of serum TG compared to the most common haplotype GTATA (41.17%) after adjusting for age, sex, BMI and waist circumference (The slope of the linear regression is 0.054, *P* = 0.042). The ATGCG haplotype (12.71%) was found to be associated with slightly higher TC level (The slope of the linear regression is 0.027, *P* = 0.030). None of the haplotypes was found to be associated with HDL-C or LDL-C.

**Table 4:** **Haplotype analysis of SNPs of *PTPN11 *on the lipid levels**

**Haplotype**	**Frequency**	**TG**		**TC**		**HDL-C**		**LDL-C**	
		**b**	***P***	**b**	***P***	**b**	***P***	**b**	***P***
GTATA	41.17%	Reference	–	Reference	–	Reference	–	Reference	–
GCGTG	27.75%	0.054	**0.042**	0.013	0.157	−0.018	0.099	0.006	0.624
GTGTA	15.26%	0.028	0.389	−0.006	0.619	−0.002	0.841	−0.017	0.265
ATGCG	12.71%	0.050	0.165	0.027	**0.030**	0.014	0.343	0.028	0.082

Differences between haplotype groups were assessed using the linear regression model adjusted for age, sex, BMI and waist circumference. *P* value in bold indicated the difference was significant comparing to the most common haplotype group (*P*<0.05).

SNPs were aligned as rs2301756, rs12423190, rs12229892, rs7958372 and rs4767860.

## Discussion

The results of our study showed that lipid profile was different between males and females that the serum TG and LDL-C levels were higher and HDL-C lower in males than in females. But no difference was observed in the level of TC. These results were similar to those of previous reports [17,18].

The associations between SNPs of *PTPN11* gene and serum lipid levels in 1003 Chinese people presented a sex-specific pattern though the distribution of genotypes had no differences between the two sexes. Rs4767860 and rs12229892 were associated with TG level in males, but these significant associations could not be observed in females. In females, the genotype AA of rs2301756 was found to be associated with higher TG compared to the most common genotype GG. The SNP of rs4767860 was associated with TC in males but no SNP was related to TC in females.

Genotypes of SNPs of *PTPN11* varied in different ethnicities. In our study, the genotypes of GG, GA and AA of rs2301756 were 75.2%, 22.4% and 2.4%, respectively. They were similar to those of Japanese (62.1%, 32.9% and 5.0%, respectively) [19] but absolutely different from those of Caucasian (0.5%, 13.2% and 86.3%, respectively) [13]. The data from Hapmap show that rs12229892 and rs4767860 are very rare or do not exist in Caucasian and African Americans while in Chinese and Japanese these two SNPs are very common. The A allele of rs12229892 was 41.7% and G allele of rs4767860 was 42.7% in our study. The C allele of rs7958372 in HapMap database is the dominant allele in Caucasian while in Asian it is the minor allele (13.4% in our study). Considering the diversity of variants of *PTPN11* in different ethnicities, the positive associations observed in our study might not be repeated in other ethnic populations.

The *PTPN11* gene, which encodes SHP2, has been reported to be associated with *helicobacter pylori*-related gastric atrophy [15,20] and gastric cancer [21]. Jamshidi et al. [13] first selected three htSNPs of *PTPN11* gene (rs2301756, rs11066320 and rs11066322) and assessed their associations with serum lipid levels in a Caucasian female population. They found that subjects with AA genotype of rs11066320 had lower LDL-C by 2.6% compared to subjects with GG genotype. They also observed a non-significant increasing trend of TG level from 1.26 mmol/L in rs11066322 GG genotype carriers to 1.47 mmol/L of AA genotype carriers. Lu et al. [22] reported that genotype AA of rs11066322 of *PTPN11* was associated with the higher plasma HDL-C levels. However, the htSNPs were different in Chinese population. One of the SNPs, rs11066320, which had MAF > 0.05 in Caucasian, did not exist in Chinese and Japanese [19]. Rs2301756 and rs11066322 were in complete disequilibrium that rs11066322 could not be chosen as htSNP. Okada et al. [19] reported that the HDL-C levels were different in the non-smokers and the current smokers within the same rs2301756 genotype, however, the role of rs2301756 was not assessed. In our study, rs2301756 was associated with TG level in females that subjects of AA genotype had higher TG than subjects of GG genotype. The mechanism underlying these associations was still in the stage of hypothesis which stated that the SNPs of *PTPN11* might change the expression of the gene and consequently influenced the protein encoded, SHP2, which could regulate lipometabolism [9,10].

Two limitations should be noted in our study. The first one was only htSNPs with MAF > 5% were studied. We could not rule out the possibility that other SNPs, especially the rare SNPs, were associated with the lipid levels, as SNPs with low minor frequency had been reported to be associated with lipid profile [23]–[26]. Sequencing of the whole gene might be the solution. The another limitation was that the influence of life style on lipid levels could not be assessed because of the design, as previous studies had reported that lifestyle factors such as cigarette or alcohol consuming could affect lipid profile [27,28]. More rigorous design would be performed in the future study.

## Conclusions

In summary, we found that SNPs of *PTPN11* gene were associated with serum lipid levels in a sex-specific pattern. Rs12229892 and rs4767860 may play an important role in lipid profile in males, and rs2301756 and rs7958372 may be related to TG and LDL-C levels in females. Further studies are needed to explore the mechanism on how *PTPN11* SNPs exert their effects on lipid profile.

## Abbreviations

SNPs: Single nucleotide polymorphisms; PTPN11: Protein tyrosine phosphatase, non-receptor 11; TG: Triglyceride; TC: Total cholesterol; HDL-C: High-density lipoprotein cholesterol; LDL-C: Low-density lipoprotein cholesterol; SHP2: Src homology-2 domain-containing protein tyrosine phosphatase 2; BMI: Body mass index; htSNPs: Haplotype tagging SNPs; MAF: Minor allele frequency; PCR: Polymerase chain reaction.

## Competing interests

The authors declare that they have no competing interests.

## Authors’ contributions

JJ and XYC designed the study. ZFJ, XYC, DHC and FK performed the experiments. ZFJ and JJ analyzed the data and wrote the first draft of manuscript. JJ and PK revised the manuscript. All authors read and approved the final manuscript.
